# Mouse experiments demonstrate differential pathogenicity and virulence of *Trypanosoma brucei rhodesiense* strains

**DOI:** 10.1016/j.exppara.2021.108135

**Published:** 2021-07-17

**Authors:** Limo William Kipkorir, Thuita Kibuthu John, Orindi Benedict Owino, Oidho John, Shivairo Robert, Masiga Daniel, Adung’a Vincent Owino

**Affiliations:** a Department of Biological Sciences, Egerton University, P. O Box, 536-20115, Egerton, Kenya; b Biotechnology Research Institute - Kenya Agricultural and Livestock Research Organisation, Chemotherapy Division, Primate Section, P.O Box, 362-00902, Kikuyu, Kenya; c Department of Biochemistry and Molecular Biology, Egerton University, P. O Box, 536-20115, Egerton, Kenya; d Department of Veterinary and Clinical Studies, Egerton University, P. O Box, 536-20115, Egerton, Kenya; e International Centre of Insect Physiology and Ecology, P. O Box, 30772-000100, Nairobi, Kenya; f Department of Animal Sciences, Meru University of Science and Technology, P.O Box, 972-60200, Meru, Kenya; g KEMRI-Wellcome Trust Research Programme, CGMRC, P. O Box, 230-80108, Kilifi, Kenya; h Department of Public Health and Primary Care, Leuven Biostatistics and Statistical Bioinformatics Centre, Kapucijnenvoer 35, Blok D, Bus 7001, B-3000, Leuven, Belgium

**Keywords:** African trypanosome, Sleeping sickness, Pathogenesis, Virulence, *Trypanosoma brucei rhodesiense*

## Abstract

*Trypanosoma brucei rhodesiense* is the causative agent for Rhodesian human African trypanosomiasis. The disease is considered acute, but varying clinical outcomes including chronic infections have been observed. The basis for these different clinical manifestations is thought to be associated with a combination of parasite and host factors. In the current study, *Trypanosoma brucei rhodesiense* strains responsible for varying infection outcomes were sought using mouse model. Clinical rHAT parasite isolates were subjected to PCR tests to confirm presence of the serum resistance associated (SRA) gene. Thereafter, four *T. b. rhodesiense* isolates were subjected to a comparative pathogenicity study using female Swiss white mice; the parasite strains were compared on the basis of parasitaemia, host survival time, clinical and postmortem biomarkers of infection severity. Isolates identified to cause acute and chronic disease were compared for establishment in insect vector, tsetse fly. The mouse survival time was significantly different (Log-rank*p* = 0.0001). With mice infected with strain KETRI 3801 exhibiting the shortest survival time (20 days) as compared to those infected with KETRI 3928 that, as controls, survived past the 60 days study period. In addition, development of anaemia was rapid in KETRI 3801 and least in KETRI 3928 infections, and followed the magnitude of survival time. Notably, hepatosplenomegaly was pronounced with longer survival. Mouse weight and feed intake reduced (KETRI 3801 > KETRI 2636 > EATRO 1762) except in KETRI 3928 infections which remained similar to controls. Comparatively, acute to chronic infection outcomes is in the order of KETRI 3801 > KETRI 2636 > EATRO 1762 > KETRI 3928, indicative of predominant role of strain dependent factors. Further, KETRI 3928 strain established better in tsetse as compared to KETRI 3801, suggesting that transmission of strains causing chronic infections could be common. In sum, we have identified *Trypanosoma brucei rhodesiense* strains that cause acute and chronic infections in mice, that will be valuable in investigating pathogen - host interactions responsible for varying disease outcomes and transmission in African trypanosomiasis.

## 1 Introduction

Sleeping sickness or human African trypanosomiasis (HAT) is a disease caused by two sub-species of *Trypanosoma brucei, T. b. rhodesiense* and *T. b. gambiense*. The parasites are transmitted during blood feeding of infected tsetse fly (*Glossina* spp), an insect vector restricted to Africa where the disease occurs. After the infective tsetse bite, the parasites multiply in the bite wound (forming chancre) and migrate to the lymphatic fluid, blood and other body tissues causing stage 1 (early or haemo-lymphatic stage) disease; stage 1 disease is characterised by malaise, bouts of fever, headaches, joint pains, itching, anaemia and lymphadenopathies (WHO, 2016; Kennedy, 2013). Thereafter, the parasites cross the blood brain barrier (BBB) into the cerebrospinal fluid (CSF) of the central nervous system (CNS), causing stage 2 (late or meningo-encephalitic stage) disease. Stage 2 disease is accompanied by neurological symptoms including confusion, sensory disturbances, incoordination and notably, disturbance of the sleep cycle from which the disease derives its name. Depending on the infecting parasite, disease progression varies; the *T. b. rhodesiense* infection (Rhodesian HAT, east African HAT) evolves rapidly in matter of weeks or months and is considered an acute disease. *T. b. Gambiense* (Gambian or west African HAT) infection results into a chronic infection that can last many months or even years. If not treated, both may result into death.

An estimated 12.3 million people in east and southern Africa live in areas where transmission of Rhodesian HAT is possible and are therefore at risk of infection (Kato et al., 2016). World Health Organization (WHO) data show that Rhodesian HAT contributed 2–5% of the annual caseload (WHO, 2016), although there are estimates that up to 40% of Rhodesian HAT cases are usually undetected (Matemba et al., 2010). Despite the general classification of Rhodesian HAT as an acute disease, a range of varying clinical outcomes have been observed (Kuepfer et al., 2011; Maclean et al., 2010); more protracted “chronic” course in some foci in south eastern Africa and more acute outcomes towards north eastern African countries (Kato et al., 2016; Mwanakasale et al., 2014; Kuepfer et al., 2011; Maclean et al., 2010) have been noted. The basis for these varying clinical manifestations is unclear, but it is thought to be associated with parasite and host genetics, and their interactions (Kato et al., 2016; Maclean et al., 2010). Importantly, the variations in clinical presentations may compromise diagnosis by lowering the index of suspicion of infection by clinical practitioners. Their occurrence therefore exacerbates an already difficult situation where diagnosis is complicated by the similarity of the clinical signs with those of other tropical diseases which can co-exist in the same localities such as malaria and typhoid fever. There is therefore a need for an improved understanding of the factors responsible for their varying clinical outcomes.

With different species and strains responsible for different disease forms and varying disease outcomes in mammalian hosts, interactions between the parasite and tsetse is also variant, and could be important in AT transmission dynamics. For example, different trypanosome species undergo their development in different fly organs (see Rotureau and Abbeele, 2013). Notably, a tsetse blood meal from infected animal does not necessarily result in fly infection, with only small population (about 5%) of tsetse flies are infected (Aksoy et al., 2003) in the wild. This number is relatively low, and various factors are implicated in establishment of infection including tsetse species, strain, age and sex (Kubi et al., 2006; Peacock et al., 2012), trypanolectins (Mauldin and Welburn, 1987; Mauldin and Weburn, 1988), tsetse immune system (Hu and Aksoy, 2006), endosymbionts (Wang et al., 2009; Weiss et al., 2013) and gut-associated EP proteins (Haines et al., 2010) among others. See Dyer et al., 2013 and Roditi and Lehane, 2008 for reviews. Presently, trypanosome factors involved remain unclear, and is of interest.

The current study investigated the hypothesis that parasite virulence/pathogenicity is a major determinant in the phenomenon of varying clinical manifestations of Rhodesian HAT. In order to investigate the role of the parasite, we used the presence of the SRA gene to confirm parasite identity of cryo-preserved isolates and subsequently used a mouse model of HAT to characterise their virulence. Furthermore, the most and least virulent *T. b. rhodesiense* isolates identified were compared for establishment in tsetse fly, *Glossina pallidipes*.

## 2 Materials and methods

### 2.1 Research ethics

The study was undertaken in adherence to experimental guidelines and procedures approved by the Institutional Animal Care and Use Committee (IACUC) of Kenya Agricultural and Livestock Research Organization — Biotechnology Research Institute (KALRO-BioRI) with approval permit Ref: C/TR/4/325/164b.

### 2.2. Swiss White Mice

Inbred Swiss white mice weighing 20–35g from KALRO-BioRI Animal Breeding Unit were used. The animals were treated for ecto- and endoparasites by subcutaneous injection with ivermectin (Ivermectin® Anupco, Ipswich, England) at a dose of 0.01mL/mouse. Donor mice immunosuppressed daily with cyclo-phosphomide at a dose of 200 mg/kg per mouse for three consecutive days were used in propagating cryo-preserved African trypanosomes prior to infection of experimental mice. The mice were maintained on a diet of commercial mice pellets (Mice pellets®, Unga Ltd, Nairobi, Kenya) and provided with clean drinking water *ad libitum* according to Ndung’u et al. (2008) and Kagira et al. (2007). The mice were also provided with wood chipping as bedding materials.

### 2.3 Tsetse Flies

To avoid any variation in susceptibility to infection that is associated with fly sex and species (Maudlin et al., 1991; Peacock et al., 2012), male teneral (0–3 day old) *Glossina pallidipes* tsetse flies obtained from KALRO-BioRI Insectary Unit were used for the study. The colony was established with pupae collected from Lambwe Valley of the western Kenya/eastern Uganda Busoga focus of HAT.

### 2.4 Trypanosome Isolates and Study Design

Trypanosome isolates identified as *T. b. rhodesiense* having been originally recovered from HAT patients in East and southern African countries of Uganda, Kenya, Tanzania, Mozambique and Botswana, were used. The isolates were selected from a collection of up to 600 cryo-stabilates at the KALRO-BioRI trypanosome bank (Murilla et al., 2014).

**Fig. 1:**
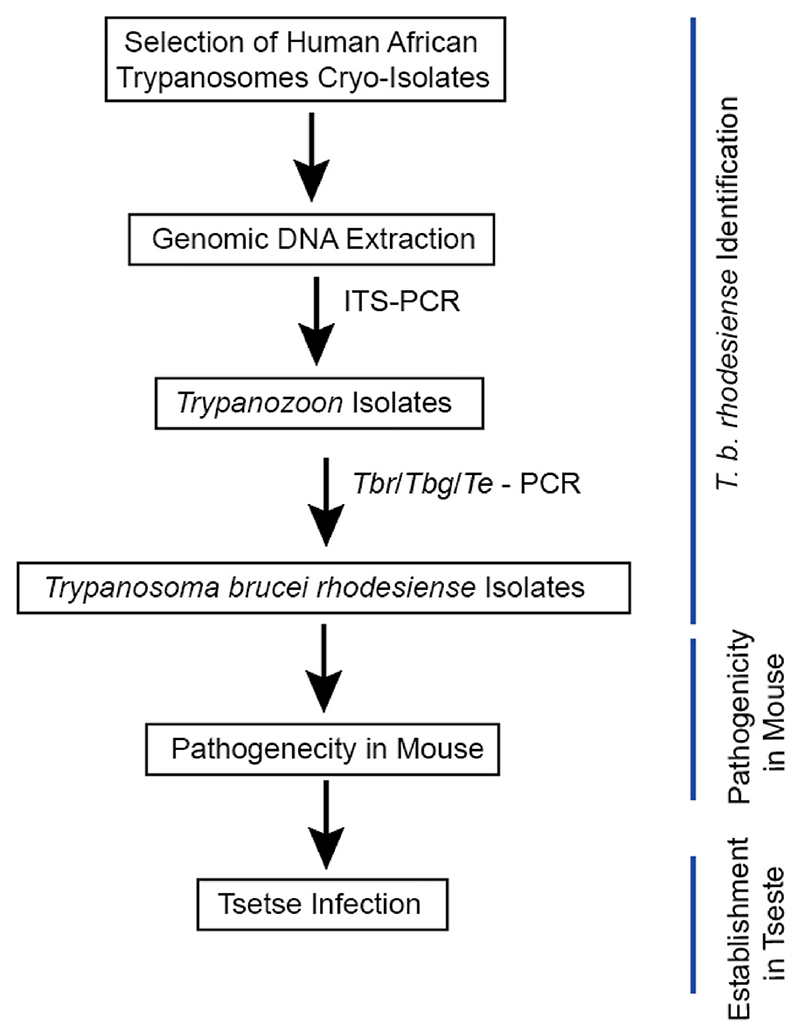
Experimental Strategy. Cryo-preserved African trypanosome isolates recovered from sleeping sickness patients were subjected to various group/species specific PCR detections to identify human infective *Trypanosoma brucei rhodesiense*. Pathogenicity/virulence studies of four selected *T. b. rhodesiense* isolates was undertaken in mouse. Consequently, the establishment of most and least virulent isolates in tsetse fly was undertaken. *Tbr: Trypanosoma brucei rhodesiense; Tbg: Trypanosoma brucei gambiense;* and *Te*: *Trypanosoma evansi*.

The isolates were confirmed as *T. b. rhodesiense* by undertaking a series of PCR amplification (see Fig. 1). Only *T. b. rhodesiense* isolates were considered further. Four confirmed *T. b. rhodesiense* strains were compared for pathogenicity and virulence in groups of Swiss white mice (n = 10). Thereafter the most virulent and least virulent isolates were compared for establishment in tsetse flies (*Glossina pallidipes*).

### 2.5 Genomic DNA Extraction and PCR Identification

Genomic DNA extraction and PCR protocols were applied for both cryo-bank isolates and tsetse organs (mouth parts - MP, salivary glands - SG and mid-guts - MG) of infected tsetse flies. Genomic DNA from cryo-isolates was extracted according to Ng’ayo et al. (2005) while from tsetse organs according to Simo et al. (2015).

The PCR reactions (see Fig. 1) were performed using appropriate primers (see table S1) as described by Njiru et al. (2005), Radwanska et al. (2002), Claes et al. (2004), Ngaira et al. (2005) and Welburn et al. (2001). HotStar HiFidelity Taq (Qiagen, Hilden Germany) was used. The products were size separated by gel electrophoresis, and captured images processed using Adobe Photoshop (Adobe Systems, California, USA).

### 2.6 Virulence and Pathogenicity Analyses

Four *T. b. rhodesiense* strains namely KETRI 3801, KETRI 2636, EATRO 1762 and KETRI 3928 were selected for pathogenicity and virulence studies using groups of inbred Swiss white mice (n = 10). KETRI 3801 and KETRI 3928 are clones previously generated using hanging drop method of Herbert and Lumsden (1976) as described by Thuita and colleagues (2008). The isolates were compared for virulence and pathogenicity by monitoring the infected mice for pre-patent period (PP), parasitaemia patterns, packed cell volume (PCV), body weight changes, feed intake, survival time up to 60 days post infection and parasite-induced changes in organ (liver and spleen) weights at post-mortem examination.

Eight (8) donor Swiss White mice were immunosuppressed as above and used to expand the selected isolates. Each thawed trypanosome cryo-stabilate was diluted in EDTA saline glucose (ESG) and intraperitoneally injected into two of the immune-suppressed Swiss white mice at 0.2 mL/mouse (1 × 104 trypanosomes). Parasitaemia levels were monitored using the matching method of Herbert and Lumsden (1976). At peak parasitaemia, typically antilog 8.1–8.4, the donor mice were deeply anaesthetised in a CO2 chamber and bled from the heart. Trypanosome count in the harvested neat blood was determined after which the harvested blood was diluted in ESG to a density of 5 × 104 trypanosomes/mL, and each experimental adult female Swiss white mice infected as above with 0.2 mL (1 × 104 trypanosomes). For each strain, 10 mice were used, with the same number used as uninfected control.

Clinical signs namely hunched appearance, lack of locomotor activity, laboured breathing, poor hair-coat condition and pallor of skin (Turner et al., 1995) were monitored on a daily basis. Parasite levels were monitored daily after day 2 post infection using matching method of Herbert and Lumsden (1976). Body weights and PCV (Cheesbrough, 2006) were recorded seven days pre-infection, on the day of infection (day 0) and after every seven days post infection. Briefly, a tail snip was performed, blood drawn from the tail into heparinized capillary tube, centrifuged at 10,000 rpm for 5 min on Haematokrit Centrifuge and their PCV read using haematocrit capillary PCV reader (Andreas Hettich GmbH & Co. KG, Tuttlingen, Germany). Consumed daily feed intake was determined by weighing feed provided after every 24 h. A post mortem was done at *extremis* or on termination of experiment, and organs (liver and spleen) weighed.

2.7

Trypanosome Establishment in Tsetse Fly:

Two strains, hyper-virulent and less virulent were used to test establishment in tsetse flies. As above, immunosuppressed mice were infected. At peak parasitaemia, typically 108 trypanosomes per mL of blood, 200 male teneral (0–3 day old) *G. pallidipes* flies per isolate were fed on infected mice. The flies were caged in groups of 25. Similarly, 200 male teneral were fed on uninfected mice and used as control. Only flies that successfully fed i.e. with engorged abdomens were retained. Unfed flies were removed from the cages and humanely euthanised. Thereafter, all the engorged flies were maintained on sterilised bovine blood.

Following successful feeding, flies in a cage were dissected per time point of 2, 4, 7, 14, 21, 28 and 42 days post feeding. Midguts (MG), salivary glands (SG) and mouth parts (MP) were examined by microscopy to detect the parasites. Subsequently, genomic DNA was extracted from both parasite positive and negative fly parts as explained above for SRA region PCR amplification.

### 2.8. Statistical Analyses

All analyses were performed using R version 3.4.3 (R Core Team, 2017). Pre-patent period (days post infection, dpi) was compared across the strains using Kruskal-Wallis test. Survival times of infected mice were estimated using Kaplan-Meier curves and the curves compared using Log-rank test (Kleinbaum and Klein, 2010). Data on PCV and body weight were compared using linear mixed effects model (LMM; Verbeke and Molenbergs, 2000) using lme4 package (Bates et al., 2015), with mouse entering the model as a random effect while strain and day — together with their interaction term — as fixed effects. Data on organ weight (spleen and liver), and feed intake were compared using a linear model with strain as the only covariate in the model, and overall strain effect assessed using F-test. To evaluate trypanosome establishment in tsetse we compared (i) the proportions of insects with the most virulent (KETRI 3801) and the least virulent (KETRI 3928) strain at day 28 post infection using Chi square test, and (ii) infected dead tsetse using logistic regression analysis, with strain and day as covariates in the model.

## 3 Results

### 3.1 PCR identification of Trypanosoma brucei rhodesiense isolates

**Fig. 2:**
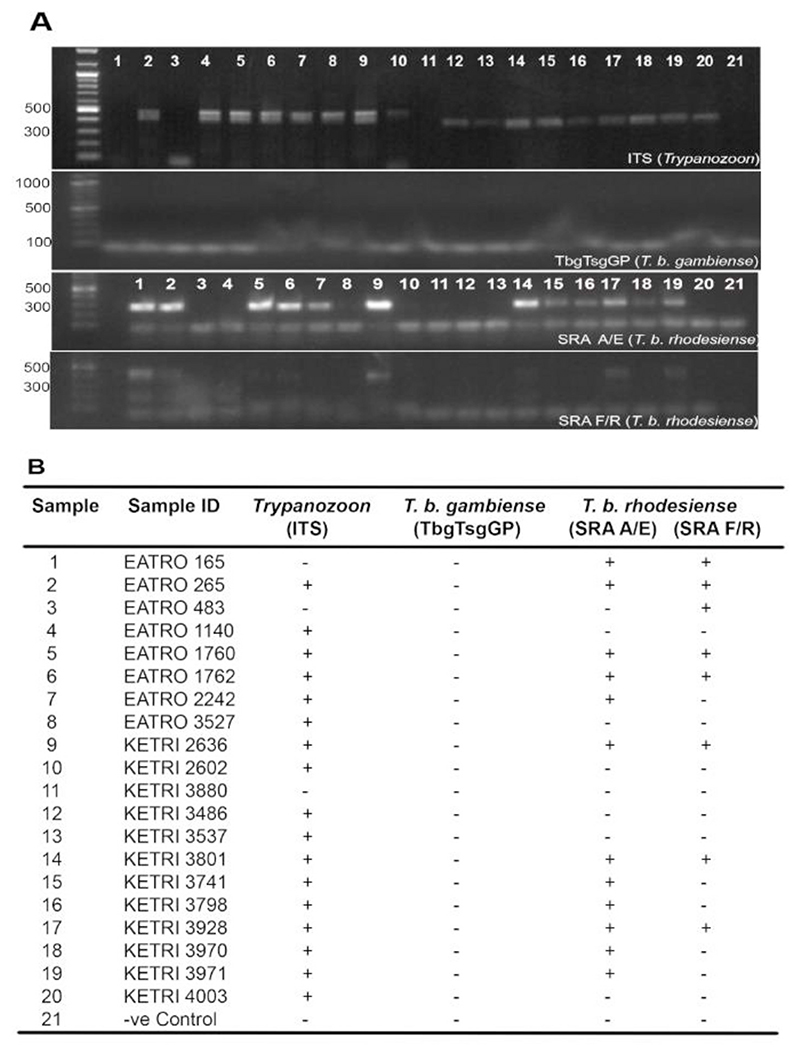
Molecular Detection of Human-Infective African Trypanosome Isolates. A. Cryo-preserved African trypanosomes recovered from patients were subjected to PCR detection using appropriate specific primers namely ITS for *Trypanozoon* group, TbgTsgGp for *Trypanosoma brucei gambiense*, and SRA A/R and F/R for *Trypanosoma brucei rhodesiense*. Most ITS positive isolates were further confirmed to be the human-infective form *T. b. rhodesiense* found in east and southern Africa and not *T. b. gambiense* found in central and western Africa. **B**. Summary of PCR detection data where + and - represent positive and negative amplifications respectively.

A total of 20 isolates were considered. Seventeen (17) were positive for *Trypanozoon* (Fig. 2) and 12 isolates had SRA gene, confirming they are *T. b. rhodesiense*, the human infective form found in East and Southern Africa. The strain EATRO 165 was negative of ITS amplification, but positive for SRA gene amplification. Of the *T. b. rhodesiense* isolates, four were selected for pathogenicity experiments in mouse-model (see Table 1). Two strains KETRI 3801 and KETRI 3928 had been earlier demonstrated to be hyper-virulent and less-virulent in vervet monkey (Thuita et al., 2008; Unpublished data). The other two selected strains were KETRI 2636 and EATRO 1762. *T. b. rhodesiense* virulence has been suggested to increase from south to north (Kuepfer et al., 2011; Kato et al., 2016; Mwanakasale et al., 2014; Kuepfer et al., 2011; Maclean et al., 2010), a factor that was applied to increase chances of selecting strains of varying virulence.

### 3.2 Comparative pathogenicity of T. b. rhodesiense isolates in mouse

**Fig. 3:**
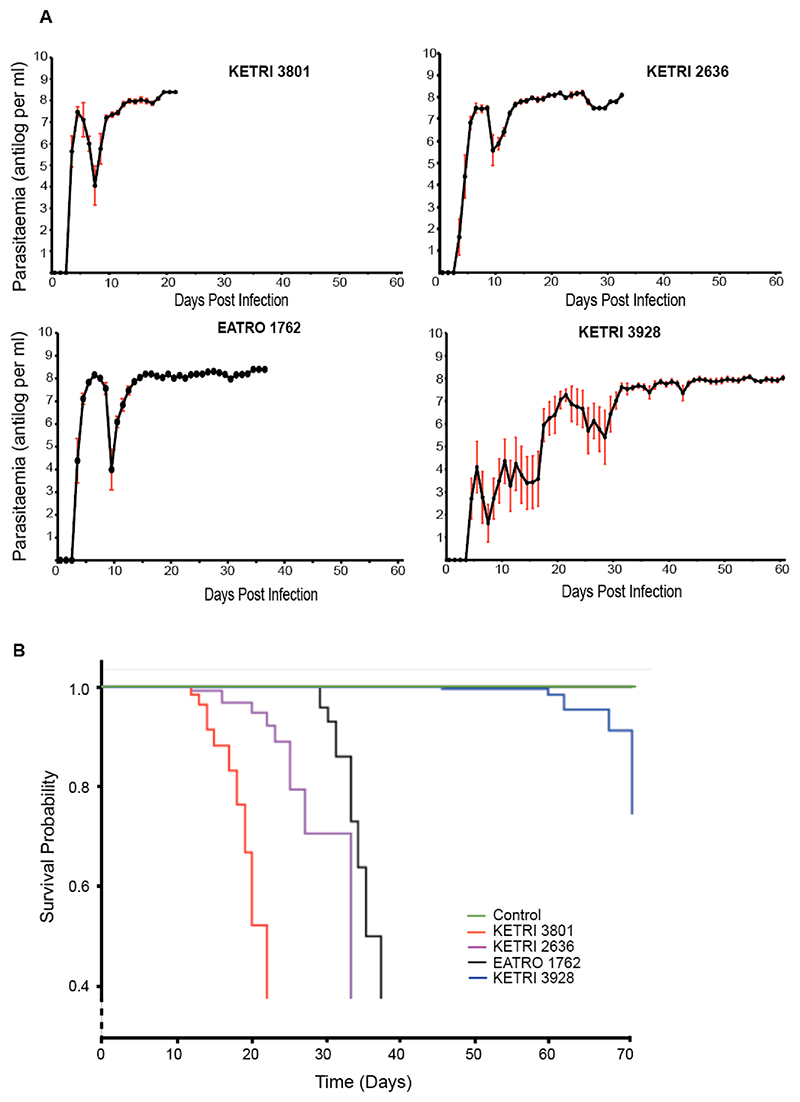
Parasitaemia Curves and Survival of Mice Infected with Various Strains of ***Trypanosoma brucei rhodesiense***. **A**. All the strains had one parasitaemia peak of antilog ≥7, and a plateau thereafter. For strain KETRI 3928, this level was reached after 20 days post infection. **B**. Survival probabilities of infected mice show KETRI 3801 as the most virulent, followed by KETRI 2636 and EATRO 1762. KETRI 3928 is the least virulent, with most mice surviving even after more than 60 days post infection.

Four *T. b. rhodesiense* isolates namely KETRI 3801, KETRI 2636, EATRO 1762 and KETRI 3928 were evaluated for virulence and pathogenicity in Swiss white mice (n = 10). The median pre-patent period (interquartile range, IQR) varied significantly from 3(3—3) days for KETRI 3801 to 4.5(4–8) days for KETRI 3928 (H = 21.4, df = 3, p = 001; Table 2). Three strains, KETRI 3801, KETRI 2636 and KETRI 1762, exhibited similar parasitaemia patterns with rapid increase in parasite levels and first parasitaemia peak (antilog ≥7) attained 4–6 days post infection (Fig. 3, Table 2) followed by a second peak and then a plateau. In contrast, the parasitaemia of KETRI 3928 remained below antilog 5.5 (or 5) for about 2 weeks — with two peaks — before finally rising to a peak level of about 7.26 on day 21 post infection (Fig. 3, Table 2), that is followed by a plateau. About 3 parasitaemia waves are observed before attaining a plateau phase in KETRI 3928.

The numbers in superscript and bracket are the last time point in days before extremis when PCV was determined.

In the course of infection, we also measured PCV, food intake, weight changes and survival times of the infected mice cohorts. Using Log-rank test, there was a significant difference in the mice survival times across the strains (Log-rank P < 0.0001) as shown in Fig. 3B. The most virulent strain was KETRI 3801, followed by KETRI 2636, EATRO 1762 and KETRI 3928; the median survival times of infected mice cohorts were 20 days, 33 days, and 35 days, respectively. Mice infected with KETRI 3928 had no much difference with the uninfected mice (control), since both cohorts never reached the median survival, and survived past the experimental period of 60 days. Notably, longer survival was not associated with parasite clearance, and high parasitaemia was observed at extremis. From these data, we infer that strain KETRI 3801 is responsible for an acute infection, while 3928 infection is chronic, with the other two falling in between. This suggests an increasing virulence of KETRI 3801> KETRI 2636>EATRO 1762 >KETRI 3928.

**Fig. 4:**
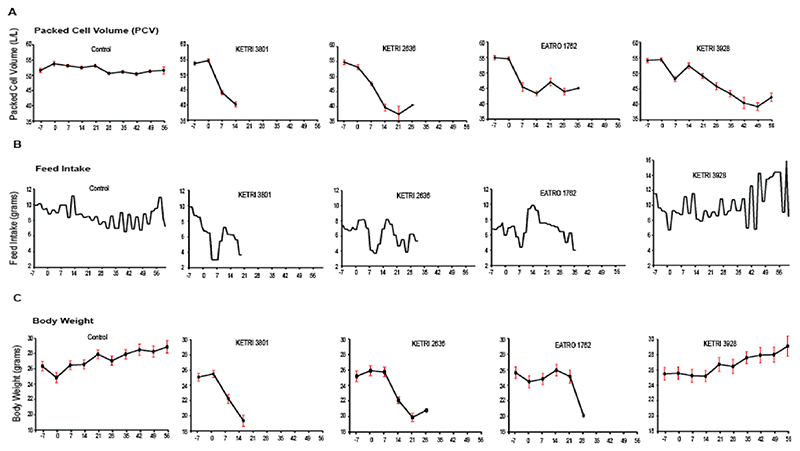
Effect of *Trypanosoma brucei rhodesiense* Strains on Mouse Packed Cell Volume (PCV), Feed Intake and Body Weight. With increasing virulence i.e. KETRI 3801 >EATRO 1726>KETRI 2636 > KETRI 3928 is increased reduction in PCV (A), feed intake (B) and body weight (C) during infection period. An exception is observed in body weight of mice infected with KETRI 3928 as compared to control which evolve in a similar pattern. In addition, feed intake fluctuations increase later on during infection as compared to control.

With varying virulence inferred from Swiss white mice survival data, we examined other clinical and pathological factors associated with the infection. The clinical signs observed include rough hair coat, reduced activity, hunched appearance, laboured breathing and crowding, that were observed within the first week of post infection with KETRI 3801 (Table 2). For KETRI 2636, EATRO 1762 and KETRI 3928, these signs were observed at about 8, 9 and 33 days post infection, respectively. PCV levels decreased during the course of infection (Fig. 4, Table 2); the highest to lowest decrease is from KETRI 3801>KETRI 2636>EATRO 1762>KETRI 3928 infections, indicative of anaemic states of the infected cohorts. Notably, (i) the rate of PCV decrease (or development of acute anaemia) is higher where survival time was lowest, and follows the magnitude of survival time; and (ii), at extremis, severe anaemia was common. Notably, in animals infected with EATRO 1762 and KETRI 3928, there is an initial PCV reduction, followed by a slight recovery and then gradual and continuous reduction. Comparing PCV reduction in KETRI 3801 and KETRI 3928, there is a drastic drop in PCV in the former, while in the later, the decrease is gradual. In all cases, the initial PCV reduction coincides with attainment of the first peak parasitaemia.

Similarly, daily feed intake for mice groups infected with KETRI 3801, KETRI 2636 and EATRO 1762 declined after infection, while the feed intake for KETRI 3928 and Control were fairly maintained throughout the entire monitoring period (Fig. 4). A similar pattern in weight loss was also observed, with KETRI 3928 infected mice not showing weight loss, but weight gain similar to uninfected mice (Fig. 4, Table 2). Comparative analyses indicate a significant difference in rate of change in both PCV (F = 49.6, DF = 4, 67.1; P < 0.0001) at 14 days post infection and body weight (F = 72.9, DF = 4, 59.6; P < 0.0001). Table 3 summarises the results for impact of strain on PCV and body weight over time (i.e. days), with highest decrease observed in the cohort infected with KETRI 3801, and least in KETRI 3928 infected cohort. This further supports our inference that KETRI 3801 is more virulent and hence responsible for acute infection, while KETRI 3928 is the least virulent strain responsible for a chronic outcome.

The post-mortem revealed that the four *T. b. rhodesiense* strains KETRI 3801, KETRI 2636, EATRO 1762 and KETRI 3928 caused four key similar pathological effects namely bilateral pneumonia, hepatosplenomegally and brain hemorrhages. Comparatively, there was significant differences across the strains for spleen weight (F = 25.78, DF = 4, 25; P < 0.0001), liver weight (F = 22, DF = 4, 31; P < 0.0001) and feed intake (F = 43.2, DF = 4, 227; P < 0.0001). See Table 4. On average, the spleen and liver weights for those infected with KETRI 3928 and EATRO 1762 were heavier than the controls, suggesting that splenomegaly and hepatomegaly is pronounced in chronic infections. Compared to control, feed intake was significantly much lower in KETRI 3801, followed by KETRI 2636 and EATRO 1762, but was significantly higher in KETRI 3928 (Table 4, Fig. 4). These models show 77.4%, 71.3% and 42.2% variability in spleen weight, liver weight and feed take, respectively.

### 3.3 Trypanosome establishment in Tsetse

**Fig. 5:**
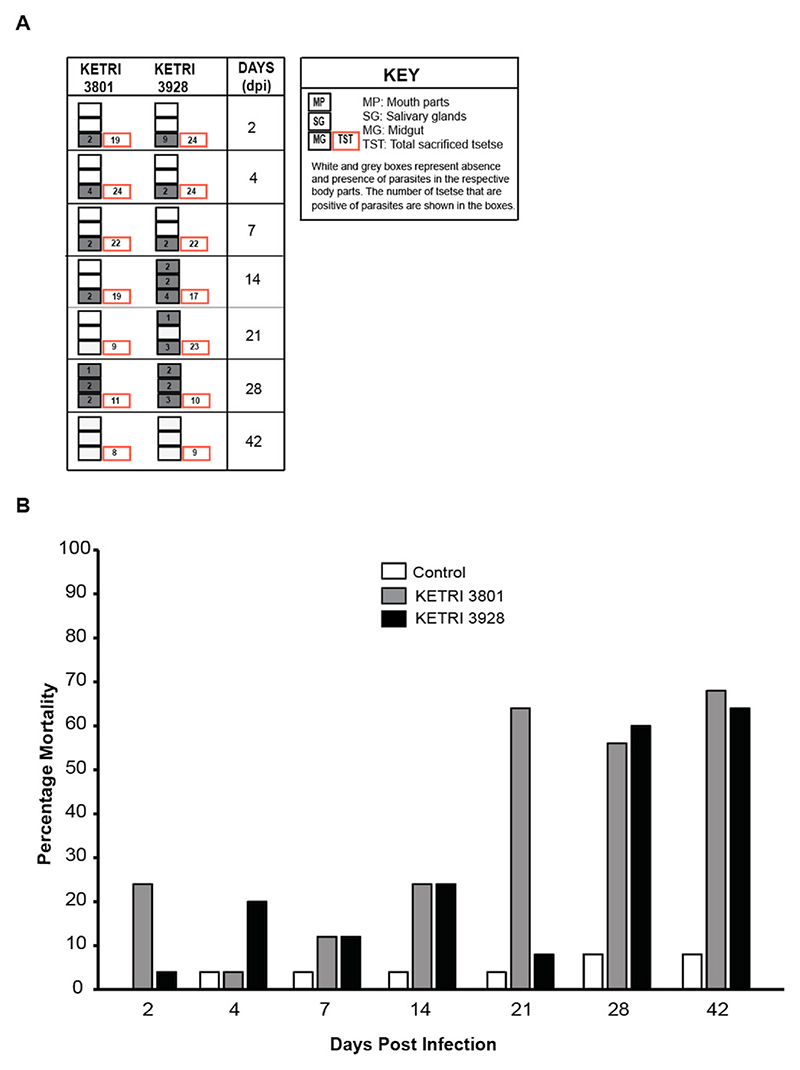
African trypanosome Insect Infection and Mortality. A. Presence of the two *Trypanosoma brucei rhodesiense* strains KETRI 3801 and KETRI 3928 of varying virulence in tsetse fly body organs namely mouth part (MP), salivary gland (SG) and midgut (MG) is shown by the grey boxes and absence by clear boxes. Total dissected tsetse are in red boxes. The infection was monitored up to day 42 after blood meal on mice infected with KETRI 3801 and KETRI 3928, and uninfected mice for control. **B**. The mortality of the infected and uninfected tsetse during the monitoring period.

We also investigated if any difference exists between strains of varying virulence in their establishment in tsetse. Therefore, over a period of 42 days, we tracked the presence of the most virulent (KETRI 3801) and least virulent (KETRI 3928) strains in different body parts of male tsetse that host the parasite during its development. Up to day 28 post infection, KETRI 3801 and 3928 were detected in 15 out of 104 (14.4%) and 32 out of 120 (26.7%) insects respectively (Chi Sq. P = 0.0375; Fig. 5A). Notable differences in number of infected insects were observed on days 14, 21 and 28 post infection. This suggests that the less virulent KETRI 3928 establishes better in tsetse as compared to KETRI 3801. In addition, high mortality was observed in infected tsetse, with highest deaths observed after two weeks post infection in both strains (Fig. 5B). Compared to control, the odds of dying for tsetse infected with KETRI 3801 and KETRI 3929 were 16.33 times and 10.83 times greater (KETRI 3801: OR = 16.33, 95%CI 7.57–39.84; KETRI 3929: OR = 10.83,95%CI 5.00–26.43) respectively. The mortality also increased days post infection (OR = 1.07, 95%CI 1.05–1.09).

## 4 Discussion

Pathogen-host interaction is complex and dynamic, with varying infection outcomes determined by host and pathogen factors, environment, and interplay between these. Environmental factors include host nutritional status, presence of other pathogens (synergistic infections and possible interactions) and previous infections (with the same pathogen or close relatives) among others. (See also Casadevall and Pirofski, 2000; Mèthot and Alizon, 2014). Host factors can be influenced by age, sex, hormonal and immune status among others. In an infection, one of the possible outcomes is disease, which manifests as damage due to perturbation of homeostasis and results into clinical signs and/or diagnostic detection of the damage (Casadevall and Pirofski, 2000); the damage is a consequence of pathogen and host responses, in the environment that the two are in. In addition, the level of damage can vary, an indication that interactions may differ between and/or in the course of infections. Our knowledge on the factors involved and their interactions is important in differentiating the range of possible outcomes such as chronic and acute, asymptomatic and latent infections, and resistance and susceptibility. In addition, it can allow us to explain cases of self-cure, alterations responsible for and transitions between latent and full blown disease, and defining virulence and pathogenicity. This knowledge can also be of practical importance in clinical diagnosis and disease management.

In this study, we show that different strains of *T. b. rhodesiense* are responsible for a range of different infection outcomes in mice, with KETRI 3801 causing acute infection, while KETRI 3928 causes chronic infection. A previous study using the vervet monkey model showed more severe pathogenic consequences in animals infected with KETRI 3801 as compared to KETRI 3928 (Thuita et al., 2008); vervet monkey is a non-human primate model of sleeping sickness (Schmidt and Sayer, 1982). In general, the different infection outcomes could be due to i) the parasite strains, ii) strain-dependent varying host responses or iii) a combination of both. While this study did not investigate the specific strain and/or host factors involved in *T. b. rhodesiense* pathogenesis and their interplay, the observed infection outcomes are likely due to strain genotype. We conclude this for several reasons. First, we used inbred Swiss White mice which are bred to minimise host variations and therefore allow for easier comparison of parasite-induced changes. Secondly, similar outcomes in mice and vervet monkey (for KETRI 3801 and 3928; see Thuita et al., 2008) infected with same strains is indicative of a strain-dependent phenomenon that is independent of the host species. Thirdly, individuals within a cohort infected with the same strains exhibit similar outcomes while cohorts infected with different strains exhibit different outcomes. If strain-dependent factors are not involved, members of cohorts infected with the same strain would exhibit different disease outcomes, which is not the case. Notably, various studies (Turner, 1990; Turner et al., 1995) demonstrated that varying virulence can be a consequence of multiple syringe passages. Here, specifically for the two strains at the end of outcome spectrum (i.e. KETRI 3801 and KETRI 3928), have less than two syringe passages, and are capable of tsetse-mediated transmission into mammalian hosts (Thuita et al., 2008). Therefore, the infection outcomes associated with them are most likely natural, host independent and not due to laboratory manipulations.

Parasite levels in the course of an infection are influenced by host-mediated clearance and parasite replication rate. The latter is dependent on doubling time and differentiation into non dividing stumpy forms in African trypanosomes. The observed reduced growth rate in KETRI 3928, in comparison to the other three strains (KETRI 3801, EATRO 1762 and KETRI 2636), has been noted as a general feature in other chronic infections (see Turner et al., 1995). Here, and considering that similar growth patterns were observed in vervet monkey (Thuita et al., 2008; Unpublished data), suggests that strain, rather than host mediated growth inhibition is a major contributor to the varying exponential growths in the early days (about 15 dpi) of infections. Differences in strain doubling time could be responsible, and also contributing to parasitaemia dependent varying virulence. Notably, a study by Tyler et al. (2001) showed that at low parasitaemia, a population of stumpy forms are present, and their numbers increase with exponential growth. This infers that increase in the population of stumpy forms could be directly proportional to replication rate. Extending this on our data, there is a possibility that the population of non-replicating stumpy forms in strain 3801 increased faster, resulting into comparatively early plateau in parasitaemia as compared to other strains. In addition, higher replication rates in 3801 and hence high densities early in infection results in accumulation of quorum-sensing derived stumpy induction factor (SIF) (Reuner et al., 1997) to threshold levels that in a density-dependent fashion triggers transformation into non replicative stumpy forms (Mony et al., 2013; Vasella et al., 1997). The density at which this occurs has been approximated to be about 106 cells/ml (McCulloch et al., 2004), that was reached by all the cell lines at different time points. This suggests that varying strain replication rates reflected by time of attaining peak parasitaemia and density dependent transformation into stumpy forms could be responsible for the varying plateau times. The presence of stumpy forms at low densities could be explained by a density-independent mechanism involving attenuation of expression site (ES) that trigger differentiation into non replicative stumpy forms that has been recently suggested (Batram et al., 2017; Zimmermann et al., 2017).

Anaemia is a known pathological feature of African trypanosomiasis, and is suggested to be due to infection-associated erythrophagocytosis and/or reduced erythropoiesis. Multiple experimental evidence suggests deleterious effects of persistent initial proinflammatory response (i.e. type 1) mediated by mononuclear phagocyte system (MPS) and other immune factors including cytokines — e.g. interferon gamma (INF-γ), interleukin 10 (IL-10), tumour necrosis factors alpha (TNF-α), interleukin 12 (IL-12) and nitric oxide (NO)— (Sileghem et al., 1994; Magez et al., 1999; Drennan et al., 2005: Magez et al., 2002) that are responsible for acute anaemia. While chronic anaemia is due to type 2 response and IL-10 mediated anti-inflammatory response. From this study, differences in anaemic condition (as inferred from PCV data) could be as a result of varying strain directed immune alterations that act in a dose dependent fashion. Here, we can speculate that elevated levels of initial type 1 immune response factors (e.g. TNF-α, INF-γ and NO) possibly contributed to acute anaemia (Naessen et al., 2005; Mabbott and Sternberg, 1995; Wu et al., 2017) in KETRI 3801 as compared to KETRI 3928 infections. For example, INF-γ whose over expression in mice results into anaemia (Cnops et al., 2015) and inhibits erythropoiesis through stem cell factor (SCF) and erythropoietin (EP) (Taniguchi et al., 1997) could be implicated. In KETRI 3928 infection however, the type 1 response is resolved, dampened by type 2 anti-inflammatory response (involving for example IL-10), resulting into chronic anaemia. In addition, extramedullary hematopoiesis in the liver and spleen could have a compensatory role, but is inadequate in the course of infection. This, and hyperactivity of these organs that is associated with infection, including erythrophagocytosis, contribute their increase in weight. The responses could be in the order of acute to chronic infection outcomes observed. Similarly, reduction in body weight could be due to loss in muscle mass as a consequence of cachexia (Taylor, 1998), a known outcome of the infection.

Clearly, the varying outcomes are most likely strain dependent, suggesting involvement of pathogen factors. These could be mediated by varying levels of parasite (strain) factors such as extracellular vesicles (EVs) and adenyl cyclase that can regulate host factors. For example, parasite EV have been implicated in induction of anaemia through erythrophagocytosis (Szempruch et al., 2016). Varying level of such parasite factors could be involved, and could be similar to varying outcomes observed in BALB/c infected with different strains of *T. brucei* (Morrison et al., 2010). This could be made clear by interrogating strain genetics and gene expression profile in the course of infection.

Management of infections that results into varying disease outcomes i.e. acute and chronic can possibly vary, with associated cost also different. In addition, chronic infection could have a huge impact in transmission dynamics of vector-borne diseases; infected mammalian hosts survive longer in chronic infections, increasing the window for a blood meal, and consequently transmission. In addition, strains responsible for chronic infections could be preferentially transmitted, if also they establish better in the transmitting vector. Here, our observation suggests better establishment of less virulent strain of *T. brucei rhodesiense* in tsetse. This could infer high transmissibility of less virulent strains, that is further enhanced by increased window for tsetse bite because of longer survival of infected mammalian hosts. One plausible reason for varying establishment could be more successful clearance of strain 3801 than 3928 by tsetse host immune response. In addition, the less virulent strain could be better adapted to survival in hosts, while the virulent strain possibly having subtle genetic changes that do not favour their association with host (both mammalian and insect). Co-evolutionary compatibilities of host and parasites could be a potential underlying factor. While these remain speculative, interrogation of interaction of these strains with tsetse at molecular level could reveal the basis of these differences. This could subsequently make clear transmission dynamics of parasites of varying virulence.

Trypanosome infected tsetse had higher mortality as compared to uninfected tsetse (Fig. 5B). We speculate that costs associated with defence against trypanosomes could negatively impact survival. Similar observation has been suggested in bumblebee - trypanosome parasite *Crithidia* system (see Moret and Schmid-Hempel, 2000; Brown et al., 2003). However, contradictory observations have been made in malaria parasites and mosquitoes (see review by Ferguson and Read, 2000). As in parasite - mammalian host interactions, varying outcomes influenced by genotypic diversity of both insects and parasites, and environments, could be expected. For vector-borne diseases, insights into this interaction will be important in understanding disease epidemiology.

In summary, we have identified two *T. b. rhodesiense* strains of varying virulence and/or that cause acute and chronic infections in mice that could be importance in understanding strain dependent pathologies of HAT. These stains could contribute to deciphering factors responsible for virulence in HAT, at molecular level. Further, they will be important in investigating vector-mediated transmission dynamics of strains responsible for varying disease outcomes.

## Supplementary Material

Supplementary data

## Figures and Tables

**Table 1:** Biological and historical data of selected *Trypanosoma brucei rhodesiense* strains.

Strain ID	Year of Isolation	Isolate Type	Region of Isolation	Lab Derivative ID for Study	Derivative Type	Passages
KETRI 3199	1989	Pleiomorphic	Busia, Kenya	KETRI 3801	Clone	1
KETRI 3928^a^	2003	Clone	Tororo, Uganda	KETRI 3928	Clone	2
KETRI 2636	1983	Pleiomorphic	Tete, Mozambique	KETRI 2636	Uncloned isolate	1
EATRO 543	1959	Pleiomorphic	Ushebu, Kahama, Tanzania	EATRO1762	Uncloned isolate	3

a A cloned strain donated to KALRO-BioRI laboratory by National Livestock Resource Research Institute (NaLIRRI) of Uganda.

**Table 2:** Parasitological and clinical effects of *Trypanosoma brucei rhodesiense* strains on mouse.

Parasitological and Clinical Parameters	Control	KETRI 3801	KETRI 2636	EATRO 1762	KETRI 3928
Median pre-patent period (IQR)	N/A	3(3–3)	4(3–5)	3(3–4)	4.5(4–8)^a^
✣First peak parasitaemia (dpi)	N/A	4	6	6	20
†Clinical signs (days)	N/A	7	8	9	33
% PCV change at day 14 dpi	−2.42	−26.51	−25.48	−20.70	−3.68
% PCV change before extremis	−4.09 (56)	−26.51 (14)	−23.95 (28)	−17.58 (35)	−22.43 (56)
% Weight change	16.21	−24.12	−19.75	−17.79	13.84
⨤Median survival time (days)	N/A	20	33	35	-

IQR: interquartile range. dpi: days post infection.

✣ First peak parasitaemia considered at level of ≥ antilog 7, and more often followed by reduction in parasite numbers.

† Clinical signs observed include rough or poor coat condition and pallor of skin, reduced activity, hunched appearance, laboured breathing and crowding.

⨤ The values are estimated from Fig. 3.

a Of the 10 mice, the pre-patent period of one animal was 17 days, and clearly an outlier.

**Table 3:** A linear mixed model analyses on comparison of changes in PCV and body weight over time on mice infection with various strains of *Trypanosoma brucei rhodesiense*.

	Packed cell volume		Body weight	
Covariate	Estimate (SE)	P value	Estimate (SE)	P value
Intercept (Control)	52.94 (0.60)	<0.001	26.01 (0.63)	<0.001
KETRI 3801	2.97 (1.04)	0.005	−1.95 (0.90)	0.035
KETRI 2636	2.59 (0.99)	0.009	−0.80 (0.90)	0.380
EATRO 1762	0.92 (0.94)	0.330	−0.83 (0.90)	0.359
KETRI 3928	1.76 (0.85)	0.039	−0.73 (0.89)	0.417
Day	−0.03 (0.02)	0.202	0.05 (0.01)	<0.001
KETRI 3801 x Day	−0.70 (0.07)	<0.001	−0.36 (0.03)	<0.001
KETRI 2636 x Day	−0.64 (0.06)	<0.001	−0.23 (0.02)	<0.001
EATRO 1762 x Day	−0.29 (0.05)	<0.001	−0.05 (0.02)	0.011
KETRI 3928 x Day	−0.22 (0.03)	<0.001	0.01 (0.02)	0.679

**Table 4:** Impact of *Trypanosoma brucei rhodesiense* strains on mouse organs (spleen and liver) weights and feed intake.

Outcome	Strain	Estimate	Std.Error	t-value	P-value
Spleen weight					
	Control (Intercept)	0.17	0.14	1.21	0.238
	KETRI 3801	0.17	0.20	0.87	0.392
	KETRI 2636	0.15	0.20	0.77	0.449
	EATRO 1762	0.66	0.18	3.70	0.001
	KETRI 3928	1.55	0.18	8.48	<0.001
Liver weight					
	Control (Intercept)	1.50	0.08	19.82	<0.001
	KETRI 3801	−0.26	0.13	−1.98	0.056
	KETRI 2636	−0.05	0.12	−0.40	0.689
	EATRO 1762	0.78	0.11	6.83	<0.001
	KETRI 3928	0.53	0.12	4.48	<0.001
Feed intake					
	Control (Intercept)	8.60	0.21	41.37	<0.001
	KETRI 3801	−2.34	0.39	−6.00	<0.001
	KETRI 2636	−2.12	0.38	−5.63	<0.001
	EATRO 1762	−1.56	0.34	−4.53	<0.001
	KETRI 3928	1.54	0.29	5.22	<0.001

## References

[R1] Aksoy S, Gibson WC, Lehane MJ (2003). Interactions between tsetse and trypanosomes with implications for the control of trypanosomiasis. Adv Parasitol.

[R2] Bates D, Maechler M, Bolker B, Walker S (2015). Fitting linear mixed-effects models using lme4. J Stat Software.

[R3] Batram C, Jones NG, Janzen CJ, Markert SM, Engstler M (2017). Expression site attenuation mechanistically links antigenic variation and development in *Trypanosoma brucei*. eLife.

[R4] Brown MJF, Loosli R, Schmid-Hempel P (2003). Condition-dependent expression of virulence in a trypanosome infecting bumblebees. Oikos.

[R5] Casadevall A, Pirofski L (2000). Host-pathogen interactions: basic concepts of microbial commensalism, colonisation, infection and disease. Infect Immun.

[R6] Cheesbrough M (2006). District Laboratory Practice in Tropical Countries.

[R7] Claes F, Radwanska M, Urakawa T, Majiwa PA, Goddeeris B, Büscher P (2004). Variable surface glycoprotein RoTat 1.2 PCR as a specific diagnostic tool for the detection of *Trypanosoma evansi* infections. Kinetoplastid Biol Dis.

[R8] Cnops J, De Trez C, Stijlemans B, Keirsse J, Kauffmann F, Barkhuizen M (2015). NK-, NKT-and CD8-derived INF_γ_ drives myeloid cell activation and erythrophagocytosis, resulting in trypanosome-associated acute anaemia. PLoS Pathog.

[R9] Drennan MB, Stijlemans B, van den Abbeele J, Quesniaux VJ, Barkhuizen M, Brombacher F (2005). The induction of a type 1 immune response following a*Trypanosoma brucei* infection is MyD88 dependent. J Immunol.

[R10] Dyer NA, Rose C, Ejeh NO, Acosta-Serrano A (2013). Flying trips: survival and maturation of trypanosome in tsetse flies. Trends Parasitol.

[R11] Haines LR, Lehane SM, Pearson TW, Lehane MJ (2010). Tsetse EP protein protects the fly midgut from trypanosome challenge. PLoS Pathog.

[R12] Herbert WJ, Lumsden WH (1976). *Trypanosoma brucei:* a rapid “matching” method for estimating the host parasitaemia. Exp Parasitol.

[R13] Hu C, Aksoy S (2006). Innate immune responses regulate trypanosome parasite infection of the tsetse fly *Glossina morsitans morsitans*. Mol Microbiol.

[R14] Kagira JM, Ngotho M, Thuita J (2007). Development of a rodent model for late stage rhodesian sleeping sickness. J Protozool Res.

[R15] Kato CD, Matovu E, Mugasa CM, Nanteza A, Alibu VP (2016). The role of cytokines in the pathogenesis and staging of *Trypanosoma brucei rhodesiense* sleeping sickness. Allergy Asthma Clin Immunol.

[R16] Kennedy PG (2013). Clinical features, diagnosis, and treatment of human African trypanosomiasis (sleeping sickness). Lancet Neurol.

[R17] Kleinbaum D, Klein M (2010). Survival Analysis.

[R18] Kubi C, van den Abbeeele J, De Deken R, Marcotty T, Dorny P, van den Bossche P (2006). The effect of starvation on the susceptibility of general and non-general tsetse flies to trypanosome infections. Med Vet Entomol.

[R19] Kuepfer I, Hhary EM, Allan M, Edielu A, Burri C, Blum JA (2011). Clinical presentation of *T. b. rhodesiense* sleeping sickness in second stage patients from Tanzania and Uganda. PLoS Neglected Trop Dis.

[R20] Mabbott N, Sternberg J (1995). Bone marrow nitric oxide production and development of anaemia in*Trypanosoma brucei*-infected mice. Infect Immun.

[R21] Maclean LM, Odiit M, Chisi JE, Kennedy PGE, Sternberg JM (2010). Focus-specific clinical profiles in human African trypanosomiasis caused by Trypanosoma brucei rhodesiense. PLoS Neglected Trop Dis.

[R22] Magez S, Stijlemans B, Caljon G, Eugster HP, De Baetselier P (2002). Control of experimental *Trypanosoma brucei* infections occurs independently of lymphotoxin-∝induction. Infect Immun.

[R23] Magez S, Radwanska M, Beschin A, Sekikawa K, De Baetselier P (1999). Tumor necrosis factor alpha is a key mediator in the regulation of experimental *Trypanosoma brucei* infections. Infect Immun.

[R24] Matemba LE, Fèvre EM, Kibona SN, Picozzi K, Cleaveland S, Shaw AP, Welburn SC (2010). Quantifying the burden of rhodesiense sleeping sickness in Urambo District, Tanzania. PLoS Neglected Trop Dis.

[R25] Maudlin I, Welburn SC (1988). The role of lectins and trypanosome genotype in the maturation of midgut infections in *Glossina morsitans*. Trop Med Parasitol.

[R26] Maudlin I, Welburn SC (1987). Lectin mediated establishment of midgut infections of *Trypanosoma congolense* and *Trypanosoma brucei* in *Glossina morsitans*. Trop Med Parasitol.

[R27] Maudlin I, Welburn SC, Milligan P (1991). Salivary gland infection: a sex-linked recessive character in tsetse?. Acta Trop.

[R28] McCulloch R, Vassella E, Burton P, Boshart M, Barry JD (2004). Transformation of monomorphic and pleomorphic*Trypanosoma brucei*. Methods Mol Biol.

[R29] Mèthot P, Alizon S (2014). What is a pathogen? Towards a process view of host-parasite interactions. Virulence.

[R30] Mony BM, Macgregor P, Ivens A, Rojas F, Cowton A, Young J (2013). Genome-wide dissection of the quorum sensing signalling pathway in Trypanosoma brucei. Nature.

[R31] Moret Y, Schmid-Hempel S (2000). Survival for immunity: the price of immune system activation for bumblebee workers. Science.

[R32] Morrison LJ, McLellan S, Sweeney L, Chan CN, MacLeod A, Tait A (2010). Role for parasite genetic diversity in differential host response to trypanosome infection. Infect Immun.

[R33] Murilla GA, Ndung’u K, Thuita JK, Gitonga PK, Kahiga DT (2014). Kenya trypanosomiasis research institute cryobank for human and animal trypanosome Isolates to support research: opportunities and challenges. PLoS Neglected Trop Dis.

[R34] Mwanakasale V, Songolo P, Babaniyi O, Simarro P (2014). Clinical presentation of human African trypanosomiasis in Zambia is linked to the existence of strains of*Trypanosoma bruceirhodesiense* with varied virulence: two case reports. J Med Case Rep.

[R35] Naessens J, Kitani H, Nakamura Y, Yagi Y, Sekikawa K, Iraqi F (2005). TNF-alpha *mediates the development of anaemia in a murine Trypanosoma brucei rhodesiense infection, but not the anaemia associated with murine Trypanosoma congolese* infection. Clin Exp Immunol.

[R36] Ndung’u K, Ngotho M, Kinyua J, Kagira J, Guya S, Ndung’u J (2008). Pathogenicity of bloodstream and cerebrospinal fluid forms of Trypanosoma brucei rhodesiense in Swiss white mice. Afr J Health Sci.

[R37] Ng’ayo MO, Njiru ZK, Kenya EU, Muluvi GM, Osir EO, Masiga DK (2005). Detection of trypanosomes in small ruminants and pigs in Western Kenya: important reservoirs in the epidemiology of sleeping sickness?. Kinetoplastid Biol Dis.

[R38] Ngaira JM, Olembo NK, Njagi EN, Ngeranwa JJ (2005). The detection of non-RoTat 1.2*Trypanosoma evansi*. Exp Parasitol.

[R39] Njiru ZK, Constantine CC, Guya S, Crowther J, Kiragu JM, Thompson RCA (2005). The use of ITS1 rDNA PCR in detecting pathogenic African trypanosomes. Parasitol Res.

[R40] Peacock L, Ferris V, Bailey M, Gibson W (2012). The influence of sex and fly species on the development of trypanosomes in tsetse flies. PLoS Neglected Trop Dis.

[R41] R Core Team (2017). R: A Language and Environment for Statistical Computing.

[R42] Radwanska M, Chamekh M, Vanhamme L, Claes F, Magez S, Magnus E, de Baetselier P (2002). The serum resistance-associated gene as a diagnostic tool for the detection of *Trypanosoma brucei rhodesiense*. Am J Trop Med Hyg.

[R43] Reuner B, Vassella E, Yutzy B, Boshart M (1997). Cell density triggers slender to stumpy differentiation of *Trypanosoma brucei* bloodstream forms in culture. Mol Biochem Parasitol.

[R44] Roditi I, Lehane MJ (2008). Interactions between trypanosome and tsetse flies. Curr Opin Microbiol.

[R45] Rotureau B, Abbeele JVD (2013). Through the dark continent: African trypanosome development in the tsetse fly. Front Cell Infect Microbiol.

[R46] Schmidt H, Sayer P (1982). *Trypanosoma brucei rhodesiense* infection in vervet monkey II. Provocation of the encephalitic late phase by treatment of infected monkeys. Tropenmed Parasitol.

[R47] Sileghem M, Flynn JN, Logan-Henfrey L, Ellis J (1994). Tumor necrosis factor production by monocytes from cattle infected with *Trypanosoma* (Duttonella) vivax and *Trypanosoma* (Nannomonas) *congolense* possible association with severity of anaemia associated with the disease. Parasite Immunol.

[R48] Simo G, Fongho P, Farikou O, Ndjeuto-Tchpuli PI, Tchouomene-Labou J, Njiokou F, Asongamyi T (2015). Trypanosome infection rate in tsetse flies in the “silent” sleeping sickness of Bafia in the centre region of Cameroon. Parasites Vectors.

[R49] Szempruch AJ, Sykes SE, Kit R, Dennison L, Becker AC, Gartrell A, Martin WJ (2016). Extracellular vesicles from *Trypanosoma brucei* mediate virulence factor transfer and cause host anemia. Cell.

[R50] Taniguchi S, Dai CH, Price JO, Krantz SB (1997). Interferon gamma downregulates stem cell factor and erythropoietin receptors but not insulin-like growth factor-I receptors in human erythroid colony-forming cells. Blood.

[R51] Taylor KA (1998). Immune responses of cattle to African trypanosomes: protective or pathogenic?. Int J Parasitol.

[R52] Thuita JK, Kagira JM, Mwangangi D, Matovu E, Turner CMR, Masiga D (2008). *Trypanosoma brucei rhodesiense* transmitted by a single tsetse fly bite in vervet monkeys as a model of human African trypanosomiasis. PLoS Neglected Trop Dis.

[R53] Turner CMR, Aslam N, Dye C (1995). Replication, differentiation, growth and the virulence of *Trypanosoma brucei* infections. Parasitology.

[R54] Turner CM (1990). The use of experimental artefacts in African trypanosome research. Parasitol Today.

[R55] Tyler KM, Higgs PG, Matthews KR, Gull K (2001). Limitation of Trypanosoma brucei parasitaemia results from density-dependent parasite differentiation and parasite killing by the hoist immune response. Proc Roy Soc Lond.

[R56] Vassella E, Reuner B, Yutzy B, Boshart M (1997). Differentiation of African trypanosomes is controlled by a density sensing mechanism which signals cell cycle arrest via the cAMP pathway. J Cell Sci.

[R57] Verbeke G, Molenberghs G (2000). Linear Mixed Models for Longitudinal Data.

[R58] Wang J, Wu Y, Yang G, Aksoy S (2009). Interactions between mutualist *Wigglesworthia* and tsetse peptidoglycan recognition protein (PGRP-LB) influence trypanosome transmission. Proc Natl Acad Sci USA.

[R59] Weiss BL, Wang J, Maltz MA, Wu Y, Aksoy S (2013). Trypanosome infection establishment in the tsetse fly gut Is influenced by microbiome-regulated host immune barriers. PLoS Pathog.

[R60] Welburn SC, Picozzi K, Fevre EM, Coleman PG, Odiit M, Carrington M, Maudlin I (2001). Identification of human-infective trypanosomes in animal reservoir of sleeping sickness in Uganda by mean of serum-resistance-associated (SRA) gene. Lancet.

[R61] World Health Organization (2016). Human african trypanosomiasis.

[R62] Wu H, Liu G, Shi M (2017). Interferon gamma in African trypanosome infections: friends or foes?. Front Immunol.

[R63] Zimmermann H, Subota I, Batram C, Kramer S, Janzen CJ, Jones NG (2017). A quorum sensing-independent path to stumpy development in Trypanosoma brucei. PLoS Pathog.

