# Supplementary material for: Mouse experiments demonstrate differential pathogenicity and virulence of Trypanosoma brucei rhodesiense strains
Source: Exp Parasitol. Author manuscript; Available in PMC 2022 Sep 7. (PMC7613321; doi:10.1016/j.exppara.2021.108135)
Supplement: Supplementary data [file EMS152540-supplement-Supplementary_data.docx]

Table S1: Primers sequences and PCR conditions.

| **Tests** | **Primer Sequences** | **Amplification Conditions** | **Reference** |
| --- | --- | --- | --- |
| ITS | CF: CCG GAA GTT CAC CGA TAT TG  BR: TTG CTG CGT TCT TCA ACG AA | Initial Denaturation: 95 ℃, 5 mins 1 cycle  Denaturation: 94 ℃, 1 min  Annealing: 58 ℃, 1 min 35 cycles  Extension: 68 ℃, 1 min  Final extension: 72 ℃ , 10 mins 1 cycle | Njiru *et al.,* 2005 |
| Tbg | TbgTgsGP-F: GCTGCTGTGTTCGGAGAGC  TbgTgsGP-R: GCCATCGTGCTTGCCGCTC | Initial Denaturation: 95 ℃, 5 mins 1 cycle  Denaturation: 94 ℃, 1 min  Annealing: 55 ℃, 1 min 35 cycles  Extension: 68 ℃, 30 sec  Final extension: 72 ℃, 10 mins 1 cycle |  |
| TE | RoTat1.2F: GCGGGGTGTTTAAAGCAATA  RoTat1.2R: ATTAGTGCTGCGTGTGTTCG  JN 2118Hu F: TTCTACCAACTGACGGAGCG  JN 2118Hu R: TAGCTCCGGATGCATCGGT | Initial Denaturation: 95 ℃, 5 mins 1 cycle  Denaturation: 94 ℃, 1 min  Annealing: 50 ℃, 1 min 35 cycles  Extension: 68 ℃, 30 sec  Final extension: 72 ℃, 10 mins 1 cycle  Initial Denaturation: 95 ℃, 5 mins 1 cycle  Denaturation: 94 ℃, 1 min  Annealing: 55 ℃, 1 min 40 cycles  Extension: 68 ℃, 30 sec  Final extension: 72 ℃, 10 mins 1 cycle | Claes *et al.,* 2004 ;  Ngaira *et al.,* 2005 |
| Tbr | SRA-F: AATGTGTTCGAGTACTTCGGTCACGCT  SRA-R:ATAGTGACAAGATGCGTACTCAACGC  B537: CCATGGCCTTTGACGAAGAGCCCG  B538: CTCGAGTTTGCTTTTCTGTATTTTTCCC | Initial Denaturation: 95 ℃, 5 mins 1 cycle  Denaturation: 94 ℃, 1 min  Annealing: 50 ℃, 1 min 35 cycles  Extension: 68 ℃, 1 min  Final extension: 72 ℃ , 10 mins 1 cycle  Initial Denaturation: 95 ℃, 5 mins 1 cycle  Denaturation: 94 ℃, 1 min  Annealing: 55 ℃, 45 sec 35 cycles  Extension: 68 ℃, 1.5 mins  Final extension: 72 ℃ , 10 mins 1 cycle | Welburn *et al.,* 2001;  Radwanska *et al.,* 2002 |
